# Skin Sceneries of Thyroid Disorders and Impact of Thyroid on Different Skin Diseases: A Scoping Review Focused on Pediatric Patients

**DOI:** 10.3390/children11121488

**Published:** 2024-12-06

**Authors:** Ignazio Cammisa, Margherita Zona, Cristina Guerriero, Clelia Cipolla, Donato Rigante

**Affiliations:** 1 Department of Life Sciences and Public Health, Fondazione Policlinico Universitario A. Gemelli IRCCS, 00168 Rome, Italy; ignazio.cammisa01@icatt.it (I.C.); margherita.zona01@icatt.it (M.Z.);; 2 Department of Dermatology, Fondazione Policlinico Universitario A. Gemelli IRCCS, 00168 Rome, Italy; 3 Università Cattolica Sacro Cuore, 00168 Rome, Italy

**Keywords:** thyroid, thyroid hormones, hyperthyroidism, hypothyroidism child, personalized medicine

## Abstract

Thyroid function plays a seminal role in the growth and development of children, and alterations in signaling pathways which interfere with the biosynthesis, transport, and metabolism of thyroid hormones might impact on the skin of such patients: this review explores the relationship between different thyroid disorders and dermatological manifestations with a particular focus on the pediatric population. Common cutaneous conditions associated with thyroid dysfunction may include chronic urticaria, vitiligo, and alopecia, which can be early harbingers of an underlying endocrine disruption. This review also highlights the growing cognizance of a “thyroid–skin axis”, with thyroid hormones influencing many physiologic processes within the skin such as keratinocyte proliferation, hair growth, and epidermal differentiation. A precocious recognition of abnormal dermatological signs can be crucial in pediatric patients for a timely diagnosis before any development of complications and for personalized treatments of an underlying thyroid disorder, which can even be symptomless at an initial phase. Despite the lack of standardized guidelines for managing dermatologic manifestations occurring in thyroid diseases, a regular screening to identify endocrine dysfunction is recommended in those children who present chronic urticaria, vitiligo, or alopecia, though further research is needed to decipher mechanisms involved in the thyroid-skin partnership and develop more targeted management approaches.

## 1. Introduction

A regular function of the thyroid is crucial for both a child’s neurodevelopment and overall somatic growth during childhood and adolescence: however, thyroid disturbances are common in the general population and careful physical examinations along with specific labwork and imaging may help in their early detection and treatment in pediatrics [1]. Identifying and treating thyroid conditions specifically in children is of paramount relevance to ensure their optimal development and standard growth, while knowing eventual risk factors, recognizing precociously any clinical signs and symptoms, and accurately interpreting the screening tests are further keys to improving diagnostic accuracy. Thyroid disorders can unequivocally impact the skin, and the most observed thyroid-related abnormalities include chronic urticaria (6.8%), vitiligo (6.8%), widespread alopecia (6%), acne vulgaris (5%), and rosacea (3.6%); specific skin symptoms can be associated with either hyperthyroidism or hypothyroidism, while some clinical pictures may be encountered in both cases [2]. The medical literature does not report peculiar differences in skin findings between autoimmune and non-autoimmune hyperthyroidism, autoimmune and non-autoimmune hypothyroidism, or autoimmune and non-autoimmune euthyroidism, but a thorough identification of skin manifestations associated with specific pathologic thyroid pictures should allow clinicians to contemplate the skin as a diagnostic handle to early detect endocrine irregularities. The main aim of this review is to provide an overview of skin manifestations occurring in thyroid dysfunctions with detailed focus on the pediatric population.

## 2. The Thyroid–Skin Axis

**Figure 1:**
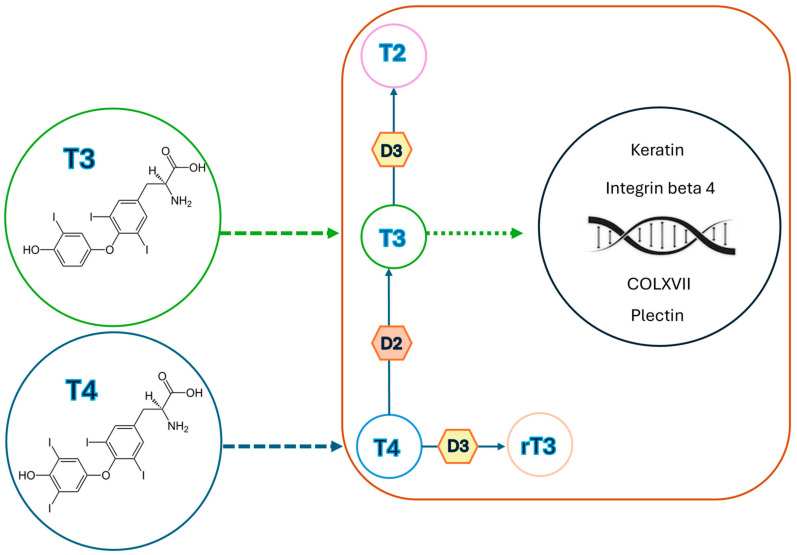
Sketch showing thyroid hormone signaling in the skin: once inside the skin cells, the type 2 and 3 deiodinases (D2 and D3) regulate thyroid hormone conversion; D2 activates T4 to T3, and D3 inactivates both T4 and T3 to rT3 and T2, respectively. Then, T3 enters into the nucleus of skin cells, modulating the expression of many genes under the influence of thyroid hormone levels, such as genes coding for keratin, integrin beta 4 (CD104), plectin, and type XVII collagen (COLXVII).

The skin, the largest organ of the body, displays remarkable functional and structural diversity, acting as a barrier between the external environment and internal milieu and being highly sensitive to various stressors as solar and thermal radiations, mechanical energy, and chemical or biological insults [3]. Moreover, in recent years, it has become increasingly clear that skin is also regulated by metabolic and neuroendocrine mediators; indeed, the thyroid–skin partnership is an emerging area of research that addresses the role of thyroid hormones (THs) in skin and their relationship with skin manifestations in case of thyroid dysfunction [4,5]. The underlying mechanisms of such skin involvement, however, are not entirely clear [6,7]. A direct influence of the thyroid gland on skin is mediated by the presence of thyroid hormone receptors (TR) on various skin cell types, influencing both expression of genes and activity of proteins, as reported in Table 1 [4,8,9]. Thyroid-stimulating hormone (TSH) expression in the epidermis is upregulated by hypothalamic thyroid-releasing hormone (TRH) and suppressed by THs, confirming that intraepidermal TSH activity is controlled by the hypothalamic–pituitary–thyroid axis [10]. THs play also a critical role in epidermis differentiation and barrier formation, stimulating the expression of specific barrier-related molecules such as involucrin and loricrin, as well as in keratinocyte proliferation and keratin gene expression, also influencing hair growth, production of glycosaminoglycans by dermal fibroblasts, and activity of sebaceous, eccrine, and apocrine glands [4,5,11]. Cianfarani et al. documented the expression of four genes, i.e., thyroid-stimulating hormone receptor (TSH-R), thyroglobulin (Tg), sodium iodide symporter (NIS), and thyroperoxidase (TPO), in human skin samples from healthy individuals [4]. They also demonstrated the ability of TSH to induce proliferation of keratinocytes and dermal fibroblasts via accumulation of intracellular cAMP in cultured cells [4]. Slominski et al. and Bodó et al. described TSH-R mRNA expression in skin biopsies, as well as in epidermal and hair follicle keratinocytes and melanocytes, dermal and hair follicle fibroblasts, and melanoma cell lines [5,11]. Furthermore, Bodó et al. detected thyrostimulin in the human epidermidis, serving as additional ligands for intra-cutaneously expressed TSH-R [11]. Additionally, Refetoff et al. and Kaplan et al. revealed the conversion of thyroxine (T4) to triiodothyronine (T3) or reverse triiodothyronine (rT3) in skin cultures, indirectly proving the local presence of TH deiodinases [10,12]. Deiodinases play a critical role in skin function by regulating the local conversion of THs, essential for various skin processes [5]. The skin expresses type 2 and type 3 iodothyronine deiodinases (DIO2 and DIO3, respectively), which catalyze different steps in the TH metabolic pathway [5]. DIO2 mediates the 5′ (outer ring) deiodination of T4 to T3, while DIO3 catalyzes the 5′ (inner ring) deiodination of both T4 and T3 to their inactive forms, rT3 and 3,3′-diiodothyronine (T2), respectively, as reported in Figure 1 [5]. These enzymes are expressed in a tissue- and phase-specific manner to regulate TH metabolism: DIO2 is predominantly found in the brain, pituitary, brown adipose tissue, heart, skeletal muscles, and thyroid, whereas DIO3 is primarily expressed in the brain, uterus, placenta, fetal membranes, and skin [5]. The combined actions of DIO2 and DIO3 constitute a cell-autonomous pre-receptor mechanism that modulates THs signaling in a time- and tissue-specific fashion, without influencing serum hormone concentrations [13]. Specifically, several studies have highlighted the importance of DIO3 in skin homeostasis, epidermal proliferation, differentiation, and maintenance of the skin barrier. Mancino et al. demonstrated that disruption of the *DIO3* gene in the epidermis severely impairs basal keratinocyte turnover, leading to thinner epidermis, altered stratification, and marked reduction in the stem cell compartment, along with decreased proliferative basal cells [13]. Furthermore, Di Cicco et al. showed, through histological and molecular analysis of skin nevi, that altered DIO3 expression is associated with increased and unbalanced cellular proliferation, suggesting that DIO3 dysfunction may contribute to skin tumors, particularly basal and squamous cell carcinoma [14]. These findings disclose non-classical functions of TRH- and TSH-mediated signaling in the skin and suggest that these hormones are not random players but significant characters in both skin physiology and human epithelial cell biology [9].

## 3. Skin Manifestations in Thyroid Diseases

### 3.1. Hyperthyroidism

Common skin symptoms of hyperthyroidism include facial flushing, erythema of the palms, and excessive sweating of both palms and soles; less frequent are generalized pruritus, eczematous dermatitis, chronic urticaria, and a general tendency to dermographism [15]. The epidermis of an overactive thyroid is thin but not atrophic, unlike that found in Cushing syndrome [15]. Changes can be also seen in the nails and hair: nails become brittler, and Plummer’s nail or distal onycholysis may also occur, though these are not specific of hyperthyroidism as they can be found in psoriasis, allergic contact dermatitis, and even hypothyroidism [16]. The hair on the scalp becomes more fragile, appearing thinner or associated with non-scarring hair loss [2]. Hyperthyroidism is usually seen in Graves’ disease (GD), and its peak incidence in the pediatric age is between 10 and 15 years [17], being more prevalent in females [17]. Skin manifestations related to GD are uncommon, affecting 1-to-4% of patients (children and adults together), and typically appear months to years after diagnosis [18]. Patients with GD may present distinctive clinical pictures such as pretibial myxedema, also known as thyroid dermopathy, and thyroid acropachy [18]. This last condition appears as a lesion on the pretibial region caused by accumulation of glycosaminoglycans, affecting less than 5% of GD patients in several forms [19,20]: the most common is the nonpitting type (43.3%), followed by plaque-like (27%), and nodular (18.5%). The rarer elephantiasic form (2.8%) is severe and linked to variable degrees of lymphatic obstruction [1]. Risk factors for thyroid acropachy include local traumas, venous stasis with subsequent edema, aberrant lymphatic circulation, and reduced clearance of fibroblast-stimulating cytokines [21,22]. The diagnosis is primarily clinical, though it should be confirmed with skin biopsy and demonstration of abnormal thyroid function tests. The histological examination usually shows dermal mucin deposits between collagen bundles, highlighted with Alcian blue and periodic acid-Schiff staining, dermal edema, and varying degrees of acanthosis and papillomatosis [23]. Clinically, it is characterized by tight skin, digital clubbing, and distinctive diaphyseal proliferation of the periosteum in the acral and distal long bones with soft tissue edema: this condition progresses over months or years and leads to gradual curving and enlargement of fingers. Regarding the pathogenesis of acropachy, it is believed that anti-TSH-R antibodies bind to TSH-R on fibroblasts in the periosteum, triggering an inflammatory response that results in cell proliferation and progressive deposition of glycosaminoglycans [24]. Diagnosis of thyroid acropachy is based on clinical and radiological findings, while the differential diagnosis should include necrobiosis lipoidica diabeticorum, sarcoidosis, lichen amyloidosis, hypoxia-associated clubbing, and pachydermoperiostosis [25]. Thyroid acropachy may follow severe cases of thyroid-related dermopathies, typically affecting the metacarpal and/or metatarsal bones of both fingers and toes, and is observed in less than 1% of patients with GD. Both pretibial myxedema and acropachy are uncommonly reported in pediatric patients [25]. These conditions can also occur in euthyroid and hypothyroid cases, as TH levels do not necessarily correlate with skin manifestations [25]. Additionally, psoriasis has been noted to be associated with increased T3 levels but not with anti-thyroid peroxidase (TPO) or anti-thyroglobulin (TG) antibody positivity and thyroid dysfunction [26]. Unfortunately, therapeutic management of these dermatological conditions remains challenging. As for pretibial myxedema, the current standard approach involves compression socks and stockings (to decrease swelling) and topical corticosteroids, commonly under occlusion; second-line therapies include intralesional or systemic corticosteroids, intralesional octreotide, pentoxifylline, high-dose intravenous immunoglobulin, plasmapheresis, surgery, and radiotherapy [27,28]. However, most of these treatments have partial success [28]. A local injection of hyaluronidase has shown some improvement in thyroid acropachy [29]. Fatourechi et al. suggested evaluating the use of biologic agents used for thyroid orbitopathy in the management of pretibial myxedema as well: these agents include tumor necrosis factor-inhibitors, the recombinant humanized anti-interleukin-6 receptor monoclonal antibody tocilizumab, tyrosine kinase inhibitors, the monoclonal anti-CD20 antibody rituximab, TSH-R-blocking antibodies, and insulin-like growth factor 1 receptor-blocking agents [30,31]. Initial approaches to solving hyperhidrosis may include topical aluminum salts (such as aluminum chloride) or iontophoresis. Systemic anticholinergic medications are helpful if topical options fail; additionally, botulinum toxin injections, liposuction, and sympathectomy may also be considered [32]. Rituximab has shown a certain efficacy in selected patients with GD showing minimal response to high dose-corticosteroids [33,34]. The dermatological signs occurring in hyperthyroidism are shown in Table 2.

### 3.2. Hypothyroidism

Hypothyroidism can either be congenital or acquired in the pediatric population. Congenital hypothyroidism is most commonly associated with thyroid agenesis, which causes a deficiency of THs during fetal development and is consequently associated with severe intellectual disability. Additionally, most affected individuals may exhibit signs of myxedema, orbital hypertelorism, a protruding abdomen with umbilical hernia, acral swelling, clavicular fat pad, and slow-growing nails [35]. Other relevant causes of hypothyroidism include Hashimoto’s thyroiditis, radiation-induced hypothyroidism following iodine-131 treatment, post-thyroidectomy hypothyroidism, and drug therapies involving lithium, bexarotene or interferons [1]. Individuals with acquired hypothyroidism often have cold, mottled, and dry skin: different studies indicate that over 80% of patients with primary hypothyroidism may show thin, rough, and hyperkeratotic epidermis, resulting in a scaly appearance [7]. Moreover, patients with hypothyroidism due to pituitary failure often exhibit fine skin wrinkling (a parchment-like skin). Additionally, excess carotene, resulting from reduced hepatic conversion of beta-carotene to vitamin A, can accumulate in the skin stratum corneum, manifesting as yellowing of the skin: this process primarily affects the palms, soles, and nasolabial fold, and the absence of scleral involvement distinguishes clinical manifestations of carotenemia from jaundice [15]. In the most severe cases of hypothyroidism, glycosaminoglycans may tend to progressively accumulate, resulting in myxedema [2]. The hands, face, pretibial region, and periorbital areas can be commonly affected, resulting in non-pitting edema; the eyes may appear puffy or swollen, the outer third of the eyebrow may be lost, and ptosis can occur due to decreased sympathetic stimulation [7]. Additional myxedema features include broadened nose, thickened lips, and macroglossia; blunted facial expressions may limit others’ ability to interpret emotions [15]. Furthermore, hypothyroidism is frequently associated with chronic urticaria, xerosis, dermatitis herpetiformis, and vitiligo. Vitiligo occurrence is significantly higher in patients with autoimmune hypothyroidism compared to patients without thyroid disorders [2]. Similar to hyperthyroidism, nails and hair can also be affected. Nails may become thin and striated, while onycholysis may be present [17,36,37,38]. Hair tends to be dry, coarse, and brittle, with a striking tendency to fall out, resulting in diffuse or partial alopecia [16,37]. Diffuse loss of scalp, body, and eyebrow hair are typical clinical signs of thyroid insufficiency [17,36,37,38]. Diffuse and partial alopecia, including genital and beard hair loss, occurs in up to 50% of patients with hypothyroidism [13]. Conversely, hypertrichosis on the back and shoulders can be indicative of hypothyroidism in children [39]. The dermatological signs occurring in hypothyroidism are listed in Table 3.

## 4. The Influence of Thyroid on Other Skin Diseases

Thyroid dysfunction, particularly the spectrum of autoimmune thyroid diseases, is associated with various skin abnormalities that may serve as indicators of more complex systemic disorders [40]. Specifically, in patients with an autoimmune thyroid disease, the function of skin cells is influenced by both changes in TH levels and presence of thyroid-specific autoantibodies [41]. Some mechanisms have been proposed to explain the association between thyroid autoimmunity and skin diseases, such as the immunomodulatory effects of anti-thyroid antibodies, a molecular mimicry between thyroid and disease-specific epitopes, and a genetic predisposition to develop anti-thyroid autoantibodies and/ or susceptibility to other autoimmune diseases. Hence, in autoimmune thyroid diseases, skin manifestations, including chronic urticaria, vitiligo and alopecia areata, may be related to either TH levels themselves or aberrant T and/or B cell activity [41]. As skin manifestations might be the first sign of thyroid dysfunction, all patients presenting with unexplained skin changes should be screened for thyropathies and subjected to regular follow-up with the aim of facilitating early diagnosis and starting treatment to finally improve their metabolic profile and overall quality of life [42].

### 4.1. Atopic Diseases

Recent studies have indicated a possible link between abnormal thyroid function and atopic diseases such as asthma, allergic rhinitis, and atopic dermatitis [42,43,44]. This connection is thought to be mediated by the immunomodulating effects of THs. In fact, they can modify the production of different cytokines contributing to the development and exacerbation of atopic diseases [43,44,45]. Additionally, autoimmune thyroid diseases such as Hashimoto’s thyroiditis and GD often co-occur with atopic diseases, suggesting a potentially common immunopathogenic network [45,46]. Regulatory T cells (T reg cells) appear to play a key-role in this setting, as they are essential for maintaining immune homeostasis and preventing autoimmune phenomena [47,48]. Lower levels of T reg cells have been demonstrated in patients with atopy [47,48]. Pedulla et al. documented an association between atopy and thyroid autoimmunity in children with atopic dermatitis, acute and chronic urticaria, and alopecia areata [49]. The authors enrolled 324 children: 187 with atopic dermatitis (AD), 95 with acute urticaria, 40 with chronic urticaria, and 2 with alopecia areata. Based on the diagnostic work-up for atopy (skin prick tests and specific IgE assay), children were divided into atopics (n = 212) and non-atopics (n = 112). In particular, they found a higher prevalence of thyroid autoimmunity (in terms of anti-TPO and/or anti-TG autoantibodies more than twice as high compared to normal reference) in atopic children than in non-atopic ones (13.67% vs. 2.67%, *p* = 0.0016) and a significant association between thyroid autoimmunity and atopy (OR = 5.76, 95% CI 1.71–19.3). A significant association was found in children with AD, for whom thyroid autoimmunity prevalence was 11.5% in atopics vs. 2.7% in non-atopics (*p* = 0.03, OR = 4.68, 95% CI 1.02–21.3). Moreover, the same authors documented that hyperthyrotropinemia in atopic children could serve as a marker of subclinical hypothyroidism, as they showed a higher prevalence of this condition in allergic and atopic children (9.9%) compared to healthy controls [50]. Consistent with these findings, Mendiratta et al. reported a higher prevalence of thyroid autoimmune phenomena (based on the presence of anti-TPO antibodies in the serum) for children with AD, with about 20% of cases exhibiting this condition. In particular, mild AD, moderate AD, and severe AD were observed in four, three, and three patients, respectively. Among these, five children had normal thyroid function tests, while the other five had abnormal results, and only one patient had symptoms indicative of a thyroid disorder [51].

### 4.2. Chronic Urticaria

Chronic urticaria (CU), a skin condition characterized by the eruption of short-lived itchy wheals lasting for more than six weeks, has been associated with autoimmune thyroid disorders, particularly in the case of chronic spontaneous urticaria [52]. Buono et al., in an observational study involving 37 children, and Levy et al., in a clinical study of 187 patients, found that the prevalence of autoimmune thyroiditis was 10 to 30 times higher in children with CU than in the general population [53,54]. Kilic et al. also reported an increased prevalence of anti-thyroid autoantibodies among patients with CU, approximately 14.8%, less than that reported in adults (14–33%) but higher than that previously reported in children [55]. Levy et al. further suggested that thyroid autoimmunity in CU is an evolving process that may manifest before, concomitantly with, or several years after the onset of urticaria. The likelihood of developing an autoimmune thyroid disease should increase with age, also emerging several years after the first appearance of CU [53]. The mechanism by which thyroid autoimmunity is associated with urticaria is poorly understood. One proposed theory suggests that anti-thyroid antibodies, which potentially might exacerbate inflammation, disrupt the physiological structure of the thyroid gland, leading to the release of different sequestered antigens: these antigens can be misrecognized as ‘non-self’ and induce subtle low-grade autoimmune responses, mediated by thyroid protein–immune complexes, which activate the classical complement pathway and lead to mast cell degranulation [55,56]. Despite this association, there are few indications for treatment with L-thyroxine in cases of CU: Dreyfus et al. reported a 9-year-old boy with severe CU that was unresponsive to antihistamines and high doses of corticosteroids. After documenting the presence of anti-thyroid antibodies, they started a treatment with levothyroxine, resulting in complete remission [57]. A physical form of CU that may occur on a familial basis with autosomal dominant inheritance is cold-induced urticaria giving rise to cryopyrin-associated periodic syndrome, a spectrum of rare complex diseases responding to anti-interleukin-1 therapy, in which thyroid involvement might occur in the background of autoinflammatory mechanisms [58]. A relevant breakthrough advance in the setting of cryopyrin-associated periodic syndrome was the introduction of biologic drugs based on the unveiled autoinflammatory nature of its symptoms and on the striking pharmacologic effects within different parts of the NLRP3 inflammasome, a key regulator of innate immunity [59,60].

### 4.3. Vitiligo

**Figure 2:**
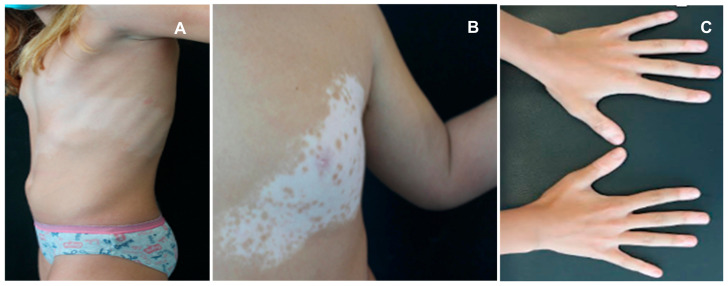
Children with thyroid abnormality associated with segmental (**A**,**B**) and non-segmental vitiligo (**C**).

Thyroid dysfunction and vitiligo are often associated in children and adolescents, with a profound impact on their physical health and mental well-being [61]. In particular, vitiligo is an acquired depigmentary disorder occurring in 50% of cases before the age of 20 and in 25% before 14 years, being characterized by a selective destruction of melanocytes in the basal layer of epidermis and sometimes in hair follicles, resulting in white patches on various parts of the body and white hair [61]. There are two main types of vitiligo: segmental (see Figure 2A,B) and non-segmental (Figure 2C). The first is characterized by a dermatomal distribution of depigmented lesions on one side of the body; in contrast, non-segmental vitiligo exhibits a chronic course often leading to widespread depigmentation, usually characterized by symmetrically located lesions. This form is the most common in children [62]. The etiopathogenesis of vitiligo is multifactorial, with the primary cause believed to be autoimmune or less frequently autoinflammatory in its origin [63,64]. Chen et al. documented a positive causal relationship between autoimmune thyroid diseases and vitiligo, corroborating a relevant number of previous studies with similar results [63,64,65,66]. The genetic susceptibility to vitiligo has been studied extensively, identifying several gene loci associated with autoimmune-based thyroid dysfunction: these include genes encoding protein tyrosine phosphatase non-receptor type 22 (or PTPN22), tyrosinase, thyroglobulin (TG), and TSH-R [65,67]. Recent observations have noted variations in the type and amount of melanocyte antigens detected in the thyroids of patients with Hashimoto’s disease, as well as the expression of thyroid-specific antigens in different skin cell types: these findings may offer a possible immunological explanation of the development of secondary vitiligo combined with thyroid dysfunction. In fact, patients with vitiligo display an overt immune activation against antigens expressed in vitiliginous melanocytes [68]. Limited data are available about the association between vitiligo and thyroid disorders in children. Kartal et al. reported that 34 out of 140 children with non-segmental vitiligo had one or more TH abnormalities (TSH, T4, T3) and anti-TPO and/or anti-TG antibody positivity, whereas none of the 15 children with segmental vitiligo had thyroid abnormalities [69]. Similarly, Iacovelli et al. documented that 10.7% of pediatric patients with vitiligo had abnormalities of thyroid function tests, while patients with segmental vitiligo showed normal results [70]. Pagovich et al. also reported that no patients with thyroid diseases had segmental vitiligo [71]. Vitiligo has been reported to come before the thyroid pathology, as reported by Zettinig et al., who found a median time of 17 years (range 4–34 years) between vitiligo start and appearance of a thyroid disease [72]. Similarly, Cho et al. reported pediatric cases of autoimmune thyroid diseases following the onset of vitiligo [73]. Screening children with vitiligo should include not only thyroid function tests but also the whole anti-thyroid antibody panel. Indeed, anti-thyroid autoantibody positivity has been found in children with vitiligo, ranging from 12 to 20%, compared to 0 to 1.9% in age-matched control groups [74]. Previous studies had shown that patients with positive anti-thyroid autoantibodies displayed a probability of extension of their vitiligo higher than 25% [75]. Therefore, children and adolescents with vitiligo, especially those with non-segmental vitiligo, should be regularly screened for thyroid dysfunction.

### 4.4. Alopecia Areata

**Figure 3:**
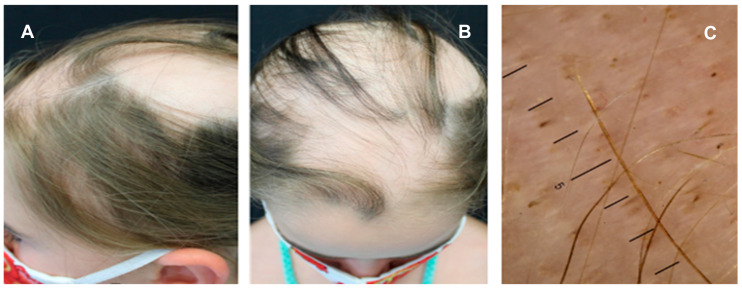
Alopecia areata with patchy and confluent hair loss (**A***,***B**) occurring on the scalp of a 7-year-old child pre senting a thyroid disease; the dermoscopic examination revealed yellow dots, black dots, and vellus hairs (**C**).

Alopecia areata (AA) is a complex genetic immune-mediated disease characterized by round or oval patches of hair loss, involving a T cell response against hair follicle self-antigens [42]. A strong association with different thyroid disorders has been described for AA, with prevalence rates ranging from 8-to-28% in adults and approximately 24% in children [18,76]. THs are crucial for a physiological growth and maintenance of hair follicles, suggesting that hair loss might represent an early clue of thyroid failure [77]. Autoimmune thyroid diseases, including Hashimoto’s thyroiditis and GD, are more frequent in adult patients with AA, who are prone to thyroid dysfunction as well as to subclinical hyperthyroidism or hypothyroidism [77]. Less is known about the relationship between AA and thyroid disorders in children. Kurtev et al. reported autoimmune thyroiditis in 47.8% of children with AA, with thyromegaly present in 66% and subclinical hypothyroidism in 15.6% of them [78]. The same authors also documented increased levels of activated CD3 + HLA-DR+ lymphocytes in patients’ peripheral blood, and this finding aligned with histological studies showing CD3 + HLA-DR+ lymphocytic infiltration in the epithelial cells of hair follicles, epidermal keratinocytes, and thyroid cells [78]. Thyroid abnormalities were also identified in 24.4% and 17.5% of children with AA by Milgraum et al. and Nanda et al., respectively [79,80]. However, Nanda et al. found a higher prevalence of thyroid abnormalities in children with extensive AA compared to those with moderate or mild disease: 30% versus 22% versus 12%, respectively [80]. A retrospective review of the National AA Registry suggested that common autoimmune disorders may occur after establishing a diagnosis of AA, highlighting the importance of ongoing surveillance for these specific patients [81]. Dermatoscopy is a valuable tool in the assessment of AA, showing empty follicles, yellow dots, dystrophic hairs, and vellus hairs [82]. Yellow dots appear as small, rounded, yellowish areas corresponding to dilated follicular openings filled with keratinous material and are observed in both acute and chronic forms [82]. Dystrophic hairs indicate the anagen phase, and are referred to as “black dots” if they appear as black punctiform areas, reflecting a premature arrest of the anagen phase before the hair emerges on the skin surface [82]. Short hypopigmented hairs are suggestive of a remission phase [82]. Figure 2 shows a female child with multiple patches of alopecia on the scalp (Figure 3A,B) who was initially treated with topical corticosteroids, then with systemic corticosteroids; the dermoscopic examination revealed the presence of yellow dots, black dots, and vellus hairs (Figure 3C).

## 5. Conclusive Remarks

The intricate relationship between thyroid function and skin tissue highlights the enlightening role of dermatological manifestations as potential early warning signs of thyroid disorders, particularly in the pediatric population. This connection is becoming increasingly recognized in clinical practice, as thyroid imbalance often presents subtly and is difficult to diagnose in children due to their nonspecific or nuanced symptoms. Dermatological manifestations, ranging from CU, vitiligo, and AA to less common conditions such as pretibial myxedema and thyroid acropachy, can serve as diagnostic handle-clues. In both hyperthyroidism and hypothyroidism, skin symptoms may be the earliest harbingers of an underlying endocrine issue, providing clinicians with the opportunity for early intervention. In pediatric patients, early diagnosis of thyroid disorders is particularly important due to their essential impact on somatic growth, cognitive development, and overall health. Conditions such as congenital hypothyroidism, autoimmune thyroiditis, and GD can have significant developmental consequences if left untreated, and the associated skin findings may help clinicians to recognize and diagnose these conditions in a precocious phase. Pediatric thyroid disorders may also overlap with autoimmune conditions, such as AD and vitiligo, suggesting a shared immunopathogenic mechanism that links the skin to thyroid activity.

Despite the growing body of evidence supporting the association between thyroid diseases and dermatological symptoms, the need for more standardized guidelines in managing these cases remains. Currently, a modern diagnostic approach involves the evaluation of thyroid function tests and screening for thyroid-specific antibodies in patients with unexplained or persistent skin conditions. However, the lack of specific treatment protocols for skin manifestations of thyroid disorders, particularly in pediatric patients, continues to be a challenge. As research progresses, it is essential that clinicians remain vigilant to the potential interplay between thyroid failure and skin pathology. Given the asymptomatic nature of many thyroid disorders in children, recognizing skin symptoms as early clinical indicators can play a pivotal role in the timely diagnosis and treatment of these endocrinologic conditions before any complications occur. Comprehensive and long-term monitoring of thyroid function in patients presenting with unusual skin conditions should become a routine part of the clinical practice. Ultimately, further research into the thyroid-skin axis may provide new insights into therapeutic strategies, improving both the quality of life and health outcomes for every patient.

## Figures and Tables

**Table 1:** Effect of thyroid hormones in the expression of human skin cells genes.

Cell Types	Involved Genes	Expression Induced by Thyroid Hormones
**Human epidermal keratinocytes**	*K6*	Increased
	*K16*	Increased
	*K17*	Increased
	*K5*	Decreased
	*K14*	Decreased
	Integrin beta 4	Decreased
	Plectin	Decreased
	*COLXVII*	Decreased
**Dermal cells**	*HAS2*	Decreased
**Human skin fibroblasts**	*AKR*	Increased
	*RAB3B*	Increased
	*COLVIA3*-*COLVIIIA1*	Increased
	*ENO1*	Increased
	*HIF*-1a	Increased
	*ZAKI* 4a	Increased
	*GLUT-1*	Increased
	*FGF7*	Decreased
	*ADH1B*	Decreased

**Table 2:** Dermatological signs occurring in hyperthyroidism.

Warm and smooth skin, excessive sweating of the palms and soles
Facial flushing, erythema of the palms
Brittle nails, Plummer’s nail and onycholysis
Fragile and thin hair on the scalp, often associated with non-scarring alopecia
Pretibial myxedema
Thyroid acropachy

**Table 3:** Dermatological signs occurring in hypothyroidism.

Dry and cold skin
Yellowing of the skin *(carotenemia)*
Protruding abdomen with umbilical hernia, acral swelling, clavicular fat pad
Chronic urticaria, xerosis, dermatitis herpetiformis and vitiligo
Striated nails and onycholysis
Alopecia, including genital and beard hair loss
Hypertrichosis on the back and shoulders

## Data Availability

Not applicable.
